# Primary Phacoemulsification and Intraocular Lens Implantation for Acute Primary Angle-Closure

**DOI:** 10.1371/journal.pone.0020056

**Published:** 2011-05-24

**Authors:** Wei-Wen Su, Phil Yeong-Fung Chen, Ching-Hsi Hsiao, Henry Shen-Lih Chen

**Affiliations:** Department of Ophthalmology, Chang Gung Memorial Hospital, Chang Gung University College of Medicine, Taoyuan, Taiwan; University of Modena and Reggio Emilia, Italy

## Abstract

**Background:**

To investigate the effect of primary phacoemulsification on intraocular pressure (IOP) in patients with acute primary angle-closure (PAC) and coexisting cataract.

**Methodology:**

Sixteen eyes of 14 patients with acute PAC received phacoemulsification and intraocular lens implantation as initial management for medically uncontrolled IOP in a retrospective chart review. The effects on IOP, vision, anterior chamber depth (ACD), and number of antiglaucoma medications were evaluated.

**Principal Findings:**

The postoperative IOP was reduced in 16 eyes (100%). The mean ± standard deviation preoperative IOP was 48.81±16.83 mm Hg, which decreased postoperatively to 16.46±10.67 mm Hg at 1 day, 9.43±3.03 mm Hg at 1 week, 9.49±2.14 mm Hg at 2 weeks, 10.78±3.56 mm Hg at 1 month, and 10.70±2.80 mm Hg at 3 months (*p*<0.001). The mean number of antiglaucoma medications decreased from 3.56±1.14 to 0.13±0.34 (*p*<0.001). The average preoperative ACD was 2.08±0.35 mm, which increased to 3.59±0.33 mm after surgery (*p*<0.001). Visual acuity (converted into logarithm of the minimum angle of resolution [logMAR]) improved from 1.14±0.71 to 0.73±0.53 (*p* = 0.001).

**Conclusions:**

Primary phacoemulsification plus intraocular lens implantation lowered IOP, reduced the use of antiglaucoma medications, and improved vision in patients with acute PAC. This is a safe and effective method of IOP control and can be considered a first treatment option in managing patients with acute PAC and coexisting cataract.

## Introduction

Acute primary angle closure (PAC) is an ophthalmic emergency that demands prompt intervention and effective treatment. Traditional treatment of acute PAC involves lowering intraocular pressure (IOP) with systemic and topical medications [1], [2]. Once the acute attack has been resolved, peripheral iridectomy or iridotomy (PI), either surgically or by laser, is usually performed to relieve pupillary block and to create a new route for aqueous humor egress. However, successful PI does not always achieve satisfactory IOP control [3]–[6].

Anterior displacement of the iris-lens diaphragm causing a shallow anterior chamber remains the major predisposing factor in acute primary angle-closure glaucoma (PACG) or PAC [7]. Eyes with angle closure tend to have a shallow anterior chamber and a thick, anteriorly positioned lens compared with normal eyes [7]–[12]. It was reported that cataract surgery widened the iridocorneal angle and attenuated the anterior positioning of the ciliary processes in eyes with PAC [13]. Recently, increased evidence has shown that primary lens extraction not only reverses the acute attack of PACG but also achieves long-term IOP control [14]–[19]. The purpose of the current study was to evaluate the effects of primary phacoemulsification and intraocular lens (IOL) implantation on IOP, vision, anterior chamber depth (ACD), and use of antiglaucoma medication in eyes with acute PAC and coexisting cataract.

## Materials and Methods

### Objective

To investigate the effect of primary phacoemulsification and IOL implantation on IOP, vision, ACD, and use of antiglaucoma medication in eyes with acute PAC and coexisting cataract.

### Participants

From January 2006 to December 2009, 16 eyes of 14 patients with acute PAC and coexisting cataract received phacoemulsification and IOL implantation as initial treatment for medically uncontrolled IOP. These patients represented part of the acute PAC cases of Chang Gung Memorial Hospital during the period under review, who visited the authors' clinic on an emergent consultation basis. The clinical diagnostic criteria for acute PAC were as follows: presentation with typical symptoms (ocular pain, blurry or halo vision, nausea or vomiting), acute increase in IOP to above 22 mm Hg, presence of ciliary flush, microcystic corneal edema, mid-dilated pupil, glaukomflecken, and occludable drainage angle. Patients with secondary glaucoma, history of uveitis, ocular trauma, previous ocular surgeries, or other ocular diseases were excluded. The enrolled patients presented visually significant intumescent cataracts which were considered to impede the normal aqueous outflow pathway and thus subject to acute IOP rise.

### Procedures

#### Initial medical treatment

After the diagnosis of acute PAC was made, these patients received first-line medical treatment for IOP control, including a combination of topical beta-blockers, brimonidine, carbonic anhydrase inhibitors (CAIs), and additional systemic hyperosmotic agents (intravenous mannitol, 1 mg/kg) and CAIs (acetazolamide, 250 mg four times daily).

#### Preoperative evaluation

The enrolled patients underwent a complete ocular examination before surgery, including best-corrected visual acuity (BCVA), IOP, keratometry, slit-lamp examination, and fundus examination. Contact A-scan biomicroscopy was performed to measure the ACD and axial length and to calculate IOL power. Visual acuity was measured with the Landolt C chart, and the value was converted into that in the logarithm of the minimum angle of resolution (logMAR).

#### Surgical procedure

After providing written informed consent, the enrolled patients underwent phacoemulsification, aspiration, and IOL implantation. All surgical procedures were performed by one surgeon (WWS). Except for one operation in which general anesthesia was used, all surgeries were performed under topical anesthesia. The surgical procedure in brief was as follows: after anesthesia and sterile procedure, a standard phacoemulsification was performed through a 2.65-mm clear corneal incision. An additional puncture was made at the limbus for chopper insertion. After injection of viscoelastic materials for anterior chamber maintenance, a continuous curvilinear capsulorrhexis (CCC) and hydrodissection were performed. The lens was phacoemulsified, and the cortical remnants were removed by irrigation and aspiration. A foldable hydrophobic acrylic IOL (SA60AT, Alcon, Fort Worth, TX) was implanted in the bag. After complete removal of viscoelastic materials, the clear corneal incision was hydrosealed or sutured with one stitch of 10/0 Nylone, depending on the final wound condition. Postoperative medication included a combination of tobramycin and dexamethasone suspension four times a day. The dosage was rapidly reduced within 1 month depending on the degree of postoperative inflammation.

#### Postoperative evaluation

Patients were followed up the morning after surgery, as well as 1 week, 2 weeks, 1 month, and 3 months postoperatively. A complete ocular examination was performed each time, including visual acuity (VA), IOP, slit-lamp examination, and fundus examination. Contact A-scan biomicroscopy was performed at the fourth visit to evaluate ACD and axial length changes.

### Ethics

The enrolled patients all provided written informed consent. The study has been approved by the institutional review board of Chang Gung Memorial Hospital.

### Statistical analysis

Continuous variables were expressed as mean ± standard deviation (SD), and categorical data were represented by number (n) and percentage (%). Variables were compared using a paired *t* test. IOP at different time points was analyzed by repeated measure one-way ANOVA. All statistical assessments were 2-sided, and a *p* level of 0.05 was considered statistically significant.

## Results

**Figure 1:**
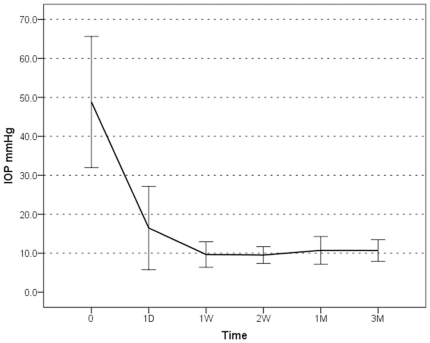
Intraocular pressure changes after phacoemulsification+intraocular lens implantation.

The mean ± SD age of the patients was 72.63±3.72 years. Five patients (35.7%) were male and nine (64.3%) were female. Most of the patients underwent surgery within 2 weeks after the acute PAC attack, with an average interval of 12.94±9.12 days. The mean IOP at the time of acute PAC attack was 48.81±16.83 mm Hg, which decreased significantly after surgery as follows: 1 day, 16.46±10.67 mm Hg, 1 week, 9.43±3.03 mm Hg, 2 weeks, 9.49±2.14 mm Hg, 1 month, 10.78±3.56 mm Hg, and 3 months, 10.70±2.80 mm Hg. A repeated measure one-way ANOVA with a Greenhouse-Geisser correction determined that IOP differed statistically significantly between time points (*p*<0.001) (Figure 1, Table 1). The mean number of antiglaucoma medications decreased from 3.56±1.41 preoperatively to 0.13±0.34 postoperatively (*p*<0.001). The preoperative BCVA (converted into logMAR) was 1.14±0.71, and the postoperative value was 0.73±0.53 (*p* = 0.001). The mean ACD increased from 2.08±0.35 mm preoperatively to 3.59±0.33 mm postoperatively (*p*<0.001). The preoperative spherical equivalent was −2.07±2.85 D, and the postoperative value was −0.25±1.16 D (*p* = 0.068). The average implanted IOL power was 22.63±2.69 D (Table 1). There were no significant postoperative complications. Transient IOP spikes were observed in two cases (12.5%).

**Table 1:** Comparison of patients' pre- and post-operative characteristics.

	Pre-OP	Post-OP	p value
IOP (mmHg)	48.81±16.83	16.46±10.67 (1 day)	*p*<0.001
		9.43±3.03 (1 week)	
		9.49±2.14 (2 weeks)	
		10.78±3.56 (1 month)	
		10.70±2.80 (3 months)	
ACD (mm)	2.08±0.35	3.59±0.33	p<0.001
Anti-glaucoma medication	3.56±1.14	0.13±0.34	p<0.001
VA (logMAR)	1.14±0.71	0.73±0.53	p = 0.001
Spherical equivalent (diopter)	−2.07±2.85	−0.25±1.16	p = 0.068
IOL power (diopter)	22.63±2.69D		

## Discussion

Iridolenticular apposition with subsequent pupillary block is a well-established etiology in PACG [7]. The iridolenticular apposition can worsen with age as the enlarging lens pushes the iris-lens diaphragm forward, narrowing the anterior chamber angle and predisposing the patient to develop angle-closure glaucoma (ACG). In the past 2 decades, an increasing number of cataract extractions had been associated with a corresponding decrease in the incidence of ACG [20]–[22]. This provides anecdotal evidence of a relationship between cataract and PACG. The “lens factor” in the pathogenesis of ACG can also be confirmed in other ways. Markowitz and Morin defined the lens thickness–to–axial length ratio (LT/AL) as a unifying index for biometric assessment of patients with ACG [23]. A greater LT/AL was strongly correlated with a shorter time before peripheral iridectomy was required [24]. Patients with a greater LT/AL were also more predisposed to acute angle-closure attack [12]. Using ultrasound biomicroscopy, Kurimoto et al. reported significant increases in ACD and trabecular-iris angle after phacoemulsification and IOL implantation, especially in eyes with a narrower anterior chamber [25]. Yang and Hung reported significant ACD increase and angle widening after extracapsular cataract extraction (ECCE) in patients with chronic PACG, using Scheimpflug image processing [26]. These findings provide evidence for lens participation in the pathogenesis of ACG and indicate beneficial effects of lens removal in PACG. Recently, increasing evidence has supported the effectiveness of primary cataract extraction in long-term IOP control, not only in patients with chronic PACG but also in those with acute PACG [14]–[19], [27]. Our results showed significantly reduced IOP, decreased use of antiglaucoma medications, and increased ACD after phacoemulsification and IOL implantation in patients with acute PAC. These results were consistent with previous reports and suggest that primary cataract extraction can be considered an alternative therapeutic option for medically uncontrolled acute PAC.

Phacoemulsification in eyes with acute PAC has unique difficulties. The cornea is usually hazy, precluding a clear view. The shallow anterior chamber provides limited room for maneuvering the nucleus, and the ultrasonic power applied must be close to the corneal endothelium. However, with the reduction of the nucleus size after several sculptings, more room is created and the anterior chamber deepens gradually, making the subsequent procedures easier. The small pupil and the atonic iris that are commonly found in post-acute-PAC eyes raise another problem. The atonic iris has a tendency to plug surgical wounds, which needs to be addressed very carefully. In managing eyes with small pupils, we gently stretch the iris with Sinski hooks bimanually to enlarge the pupil size before CCC and phacoemulsification, or use iris retractors as needed. None of the cases in our series demonstrated significant corneal decompensation during postoperative follow-up, and this surgically related complication is acceptable.

The eyes in our series underwent primary phacoemulsification without previous iridotomy. Iridectomy, performed either by laser or surgically, has been a classic treatment for PACG. Iridotomy relieves pupillary block by creating a new route for aqueous humor to egress from the posterior to the anterior chamber. However, a patent iridotomy does not always achieve satisfactory IOP control, and its effect may also diminish with time [3]–[6]. Jacobi et al. compared the effect on IOP of primary phacoemulsification and of conventional surgical iridectomy in patients with acute PACG. Their report showed better IOP control in the phacoemulsification group. Surgical iridectomy was effective in reducing IOP initially but was associated with multiple surgical reinterventions [16]. In a randomized trial, Lam et al. reported that early phacoemulsification (1 month after the abortion of the acute PAC) was more effective in preventing IOP increase than laser peripheral iridotomy in patients with acute PAC [6]. These results confirmed that lens extraction is better than iridectomy for uncontrolled PACG. In our series, most patients received primary phacoemulsification within 2 weeks after the acute PAC attack, suggesting that early phacoemulsification alone can achieve good IOP control in patients with medically uncontrolled acute PAC, with limited complications. However, besides the optimal timing of surgery, we suggest that the treatment plane be individualized depending on the patient's age, glaucoma stage, general health condition, and other risk factors.

### Conclusion

Primary phacoemulsification plus IOL implantation is a safe and effective method of IOP control for patients with acute PAC. This procedure alone can be considered a first treatment of choice for patients with medically uncontrolled acute PAC and coexisting cataract.
